# A case of lung metastasis from gastric cancer presenting as ground-glass opacity dominant nodules

**DOI:** 10.1186/s13019-024-02860-2

**Published:** 2024-06-24

**Authors:** Takahiro Niimi, Joji Samejima, Yutaro Koike, Tomohiro Miyoshi, Kenta Tane, Keiju Aokage, Tetsuro Taki, Genichiro Ishii, Masahiro Tsuboi

**Affiliations:** 1 Department of Thoracic Surgery, National Cancer Center Hospital East, Kashiwa, Chiba Japan; 2 Department of Pathology and Clinical Laboratories, National Cancer Center Hospital East, Kashiwa, Chiba Japan; 3 National Cancer Center Hospital East, National Cancer Center, 6-5-1, Kashiwanoha, Kashiwa, Chiba 277-8577 Japan

**Keywords:** Metastatic pulmonary tumor, Ground-glass opacity, Gastric cancer, Signet ring cell, Tumor volume-doubling time

## Abstract

**Background:**

Most metastatic lung tumors present as solid nodules on chest computed tomography (CT). In contrast, ground-glass opacity on chest computed tomography usually suggests low-grade malignant lesions such as adenocarcinoma in situ or atypical adenomatous hyperplasia of the lung.

**Case presentation:**

A 75-year-old woman with a history of gastric cancer surgery approximately 5 years prior was referred to the Department of Thoracic Surgery at our hospital because of two newly appearing pulmonary ground-glass opacity-dominant nodules on chest computed tomography. She had two ground-glass opacities in the right lower lobe, one in the S6 segment was 12 mm and the other in the S10 segment was 8 mm. On chest computed tomography 15 months prior to referral, the lesion in the S6 segment was 8 mm, and the lesion in the S10 segment was 2 mm. She was suspected to have primary lung cancer and underwent wide-wedge resection of the nodule in the S6 segment. In the resected specimen, polygonal tumor cells infiltrated the alveolar septa, with some tumor cells exhibiting signet ring cell morphology. Based on morphological similarities to the tumor cells of previous gastric cancers and the results of immunostaining, the patient was diagnosed with lung metastases of gastric cancer.

**Conclusions:**

Pulmonary nodules in patients with a history of cancer in other organs, even if ground-glass opacity is predominant, should also be considered for the possibility of metastatic pulmonary tumors if they are growing rapidly.

## Background

Most metastatic lung tumors present as consolidation-component-dominant nodules on chest computed tomography (CT). In contrast, ground-glass opacity (GGO) on chest CT usually suggests low-grade malignant lesions, such as adenocarcinoma in situ (AIS) or atypical adenomatous hyperplasia (AAH) of the lung.

Here, we report a rare case of lung metastasis from gastric cancer presenting as GGO-component-predominant nodules on chest thin-section computed tomography (CT).

## Case presentation

**Fig. 1:**
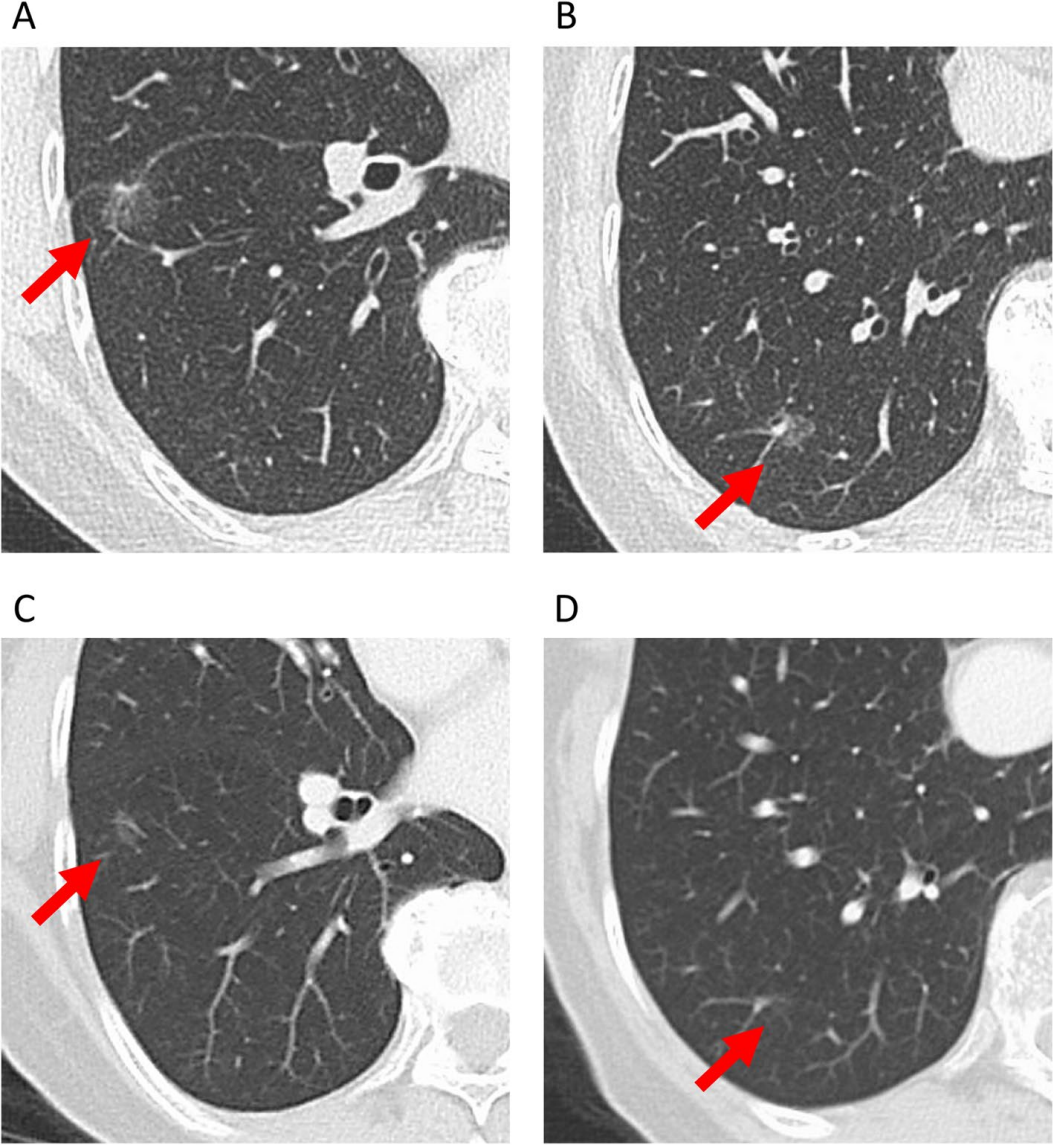
Chest CT findings. **A** Preoperative CT finding of the nodule in S6 segment. **B** Preoperative CT finding of the nodule in S10 segment. **C** Finding of the nodule in S6 segment on chest CT 15 months before referral. **D** Finding of the nodule in S6 segment on chest CT 15 months before referral

**Fig. 2:**
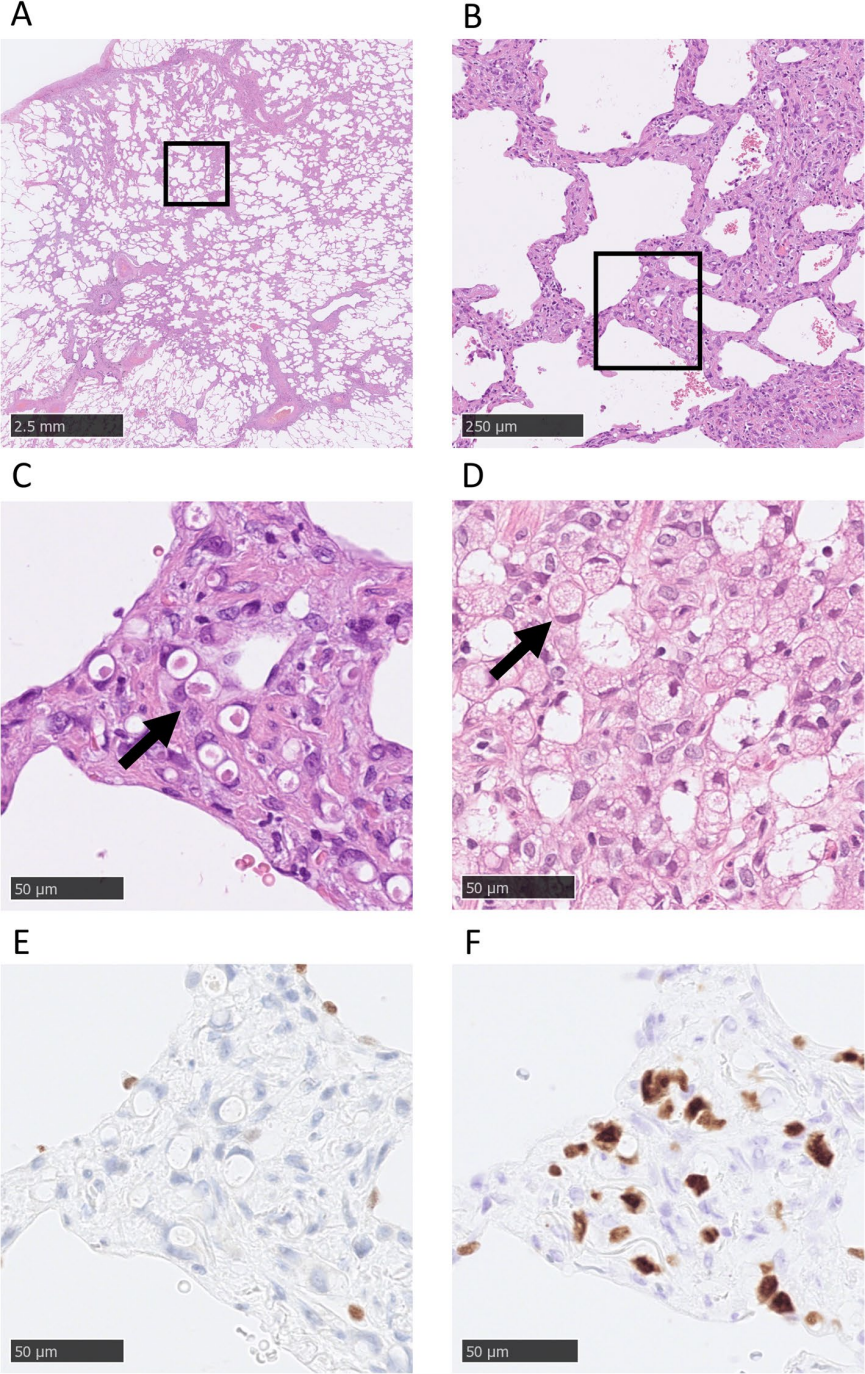
Pathological findings of resected specimen. **A** Low power view of HE staining. **B** Middle power view of HE staining. **C** High power view of HE staining. **D** Pathological finding of previous gastric cancer. **E** TTF-1 immunostaining. **F** HNF4α immunostaining

A 75-year-old woman was referred to the Department of Thoracic Surgery because of two newly appearing pulmonary nodules on her chest CT. She underwent laparoscopic distal gastrectomy with D2 lymphadenectomy for gastric cancer at 70 years old. The final pathological diagnosis was poorly differentiated adenocarcinoma with partial signet-ring cell morphology, pT1bN1M0 Stage IB (the 8th edition of the Tumor-Node-Metastasis classification of gastric cancer published by the Union for International Cancer Control (UICC)), which was identified on a follow-up CT of gastric cancer 61 months after gastrectomy. She had a history of percutaneous coronary intervention for angina pectoris and was prescribed aspirin. Tumor markers including carbohydrate antigen 19–9, carcinoembryonic antigen, squamous cell carcinoma-related antigen, cytokeratin 19 fragment, pro-gastrin-releasing peptide, and neuron-specific enolase were negative. Thin-section chest computed tomography revealed two pulmonary nodules in the S6 and S10 segments of the right lower lobe of the lung. The nodule in the S6 segment was a part-solid nodule with a maximum tumor diameter of 12 mm and a consolidation component diameter of 3 mm, whereas the nodule in the S10 segment was an 8 mm pure GGO (Fig. 1A, B). Synchronous primary lung cancer was suspected. The nodule in the S6 segment was 8 mm, and the nodule in the S10 segment was 2 mm on CT 15 months before referral (Fig. 1C, D). The nodule in the S10 segment was a pure GGO and was suspected to be very early-stage lung cancer. Therefore, we decided to perform only wide-wedge resection for the nodule in the S6 segment and to follow up the nodule in the S10 segment with CT and treat it when it became larger. She underwent wide-wedge resection for the right lower lobe of the lung. In the resected specimen, polygonal tumor cells infiltrated the alveolar septa, with some tumor cells having a signet-ring cell morphology (Fig. 2A, B and C), and they were similar to previous gastric cancer cells (Fig. 2D). These cells were negative for thyroid transcription factor 1 (TTF-1) and positive for hepatocyte nuclear factor 4 alpha (HNF4α) (Fig. 2E, F). Based on the similarity in morphology with tumor cells from a previous gastric cancer case and the immunostaining results, the final diagnosis was pulmonary metastasis from gastric cancer. Although the nodule in the S10 segment was also suspected to be a lung metastasis from gastric cancer, the patient refused chemotherapy and preferred follow-up.

## Discussion and conclusions

The lungs are common target organs for metastatic tumors. Generally, metastatic lung tumors present as single or multiple pure solid nodules on chest CT scans. GGO on chest CT are often a finding of inflammatory disease, pulmonary edema, AAH, or AIS in the lungs. In our case, a lung metastatic tumor from gastric cancer presented as a GGO-dominant pulmonary nodule on a thin-section chest CT. Although it is unusual for metastatic lung tumors to present with GGO on CT, there have been several reports of lung metastases from melanoma, thyroid carcinoma, pancreatic cancer, cholangiocarcinoma, malignant phyllodes tumor, sarcoma, gastric cancer, breast cancer, and malignant schwannoma presenting with GGO [1–14]. Metastatic lung tumors exhibit GGO on chest CT by two mechanisms. First, tumor cells grow to replace the alveolar epithelium, and the alveolar structure is preserved, similar to the AIS of the lung. It has been previously reported that pulmonary metastases of melanoma, thyroid carcinoma, pancreatic cancer, and cholangiocarcinoma exhibit this form of proliferation [1–8]. Second, tumor cells infiltrate and proliferate mainly within the alveolar septum, with only slight destruction of alveolar structures. Pulmonary metastasis from malignant phyllodes tumors and sarcomas has been reported to involve this form of proliferation [9, 10]. In addition, there have been a few reports of pulmonary metastases from gastric signet-ring cell carcinoma presenting with GGO on chest CT through this form of extension [11, 12]. Based on the pathological findings, our case was considered to present as GGO on chest CT by a second mechanism, similar to the previously reported pulmonary metastasis from gastric cancer. However, in previous reports, the pulmonary metastatic lesions were diffusely present, just like interstitial pneumonia, and there are no reports of a few isolated nodules like this case. In addition, previous reports have not mentioned changes in size over time.

In this case, the tumor volume-doubling time of the nodule in S6 was 259 days. In previous reports, the median tumor volume-doubling time of GGO in primary lung cancer was reported 400–1800 days [15–18], and the nodule in the S6 segment in our case grew faster than the typical GGO in primary lung cancer. Because of its unusually rapid growth and previous history of gastric signet-ring cell carcinoma, we might have considered lung metastasis of gastric cancer as a differential diagnosis.

To the best of our knowledge, this is the first report to demonstrate the growth rate of GGO in lung metastases from gastric cancer. Even if the nodule is GGO-dominant on the chest CT, a metastatic lung tumor should be considered as a differential diagnosis if it is growing rapidly and the patient has a history of cancer in other organs.

## Data Availability

No datasets were generated or analysed during the current study.

## Abbreviations

CT: Computed tomography
GGO: Ground-glass opacity
AIS: Adenocarcinoma in situ
AAH: Atypical adenomatous hyperplasia
TTF-1: Thyroid transcription factor 1
HNF4α: Hepatocyte nuclear factor 4 alpha

## References

[1] Borghesi A, Tironi A, Michelini S, Scrimieri A, Benetti D, Maroldi R (2019). Two synchronous lung metastases from malignant melanoma: the same patient but different morphological patterns. Eur J Radiol Open.

[2] Dalpiaz G, Asioli S, Fanti S, Rea G, Marchiori E (2018). Rapidly growing pulmonary ground-glass nodule caused by metastatic melanoma lacking uptake on 18F-FDG PET-CT. J Bras Pneumol.

[3] Mizuuchi H, Suda K, Kitahara H, Shimamatsu S, Kohno M, Okamoto T, Maehara Y (2015). Solitary pulmonary metastasis from malignant melanoma of the bulbar conjunctiva presenting as a pulmonary ground glass nodule: report of a case. Thorac Cancer.

[4] Kang MJ, Kim MA, Park CM, Lee CH, Goo JM, Lee HJ (2010). Ground-glass nodules found in two patients with malignant melanomas: different growth rate and different histology. Clin Imaging..

[5] Okita R, Yamashita M, Nakata M, Teramoto N, Bessho A, Mogami H (2005). Multiple ground-glass opacity in metastasis of malignant melanoma diagnosed by lung biopsy. Ann Thorac Surg.

[6] Ryuko T, Sano Y, Kitazawa R, Otani S, Sakao N, Mori Y (2022). Lung metastasis from thyroid carcinoma showing a pure ground-glass nodule. Ann Thorac Surg.

[7] Aissaoui M, Lupo A, Coriat R, Terris B, Bennani S, Chassagnon G, Revel MP (2021). CT features of lung metastases from pancreatic adenocarcinoma: Correlation with histopathologic findings. Diagn Interv Imaging.

[8] Nagayoshi Y, Yamamoto K, Hashimoto S, Hisatomi K, Doi S, Nagashima S, Kurohama H, Ito M, Takazono T, Nakamura S, Miyazaki T, Kohno S (2016). An autopsy case of Lepidic pulmonary metastasis from Cholangiocarcinoma. Intern Med.

[9] Nakamura S, Goto T, Nara S, Kawahara Y, Yashiro S, Kano S, Hosokawa Y, Kamada H (2020). Pure ground glass opacity (GGO) on chest CT: a rare presentation of lung metastasis of malignant phyllodes tumor. Breast Cancer.

[10] Welter S, Grabellus F, Bauer S, Schuler M, Eberhardt W, Tötsch M, Stamatis G (2012). Growth patterns of lung metastases from sarcoma: prognostic and surgical implications from histology. Interact Cardiovasc Thorac Surg.

[11] Abe Y, Suzuki M, Tsuji K, Sato M, Kimura H, Kimura H, Nagaoka K, Takakuwa E, Matsuno Y, Konno S (2020). Lung metastasis from gastric cancer presenting as diffuse ground-glass opacities. Respir Med Case Rep.

[12] Kundu S, Murphy J, Towers M, Leung CS (1999). Computed tomographic demonstration of very-low-density pulmonary nodules in metastatic gastric carcinoma: case report. Can Assoc Radiol J.

[13] Kim SB, Lee S, Koh MJ, Lee IS, Moon CS, Jung SM, Kang YA (2013). Ground-glass opacity in lung metastasis from breast cancer: a case report. Tuberc Respir Dis (Seoul).

[14] Borghesi A, Bercich L, Michelini S, Bertagna F, Scrimieri A, Maroldi R (2019). Pulmonary metastases from malignant epithelioid schwannoma of the arm presenting as fast-growing subsolid nodules: Report of an unusual case. Eur J Radiol Open.

[15] Chang B, Hwang JH, Choi YH, Chung MP, Kim H, Kwon OJ, Lee HY, Lee KS, Shim YM, Han J, Um SW (2013). Natural history of pure ground-glass opacity lung nodules detected by low-dose CT scan. Chest.

[16] Lee SW, Leem CS, Kim TJ, Lee KW, Chung JH, Jheon S, Lee JH, Lee CT (2013). The long-term course of ground-glass opacities detected on thin-section computed tomography. Respir Med.

[17] Qi LL, Wu BT, Tang W, Zhou LN, Huang Y, Zhao SJ, Liu L, Li M, Zhang L, Feng SC, Hou DH, Zhou Z, Li XL, Wang YZ, Wu N, Wang JW (2020). Long-term follow-up of persistent pulmonary pure ground-glass nodules with deep learning-assisted nodule segmentation. Eur Radiol.

[18] Song YS, Park CM, Park SJ, Lee SM, Jeon YK, Goo JM (2014). Volume and mass doubling times of persistent pulmonary subsolid nodules detected in patients without known malignancy. Radiology.

